# Bilateral Tibial Tubercle Sleeve Fractures in a Skeletally Immature Patient

**DOI:** 10.1155/2013/969405

**Published:** 2013-04-03

**Authors:** Rasesh R. Desai, Shital N. Parikh

**Affiliations:** ^1^Orthopaedic Surgeon, Southwest Regional Medical Center, 425 Home Street, Georgetown, OH 45121, USA; ^2^Department of Orthopaedic Surgery, Cincinnati Children's Hospital Medical Center, 3333 Burnet Avenue, Cincinnati, OH 45229, USA

## Abstract

Tibial tubercle sleeve fracture is a rare injury. In concept, it is similar to the patellar sleeve fracture in a skeletally immature patient. We describe a unique case of simultaneous bilateral tibial tubercle sleeve fractures in a 12-year-old boy. Radiographs and MRI confirmed the injury. The patient underwent open surgical repair of bilateral sleeve fractures with suture anchor fixation. At the final followup, 3 years after his initial injury, the patient demonstrated full knee function bilaterally without radiographic evidence of growth disturbances.

## 1. Introduction

In 2002, Davidson and Letts [1] first reported on 3 children with metaphyseal sleeve fractures of the tibial tubercle characterized by avulsion of a large area of periosteal attachment of the patellar tendon associated with small subchondral fragments of bone. This injury pattern of tibial tubercle sleeve fracture is similar to the well-recognized sleeve avulsion fracture pattern of the patella in children [2, 3]. We report a case of simultaneous bilateral tibial tubercle sleeve fractures in a skeletally immature patient. The recognition of this injury pattern would help raise awareness regarding its diagnosis and management. The patient's family gave their informed consent for pertinent information related to his injury to be submitted for publication.

## 2. Case Report

**Figure 1:**
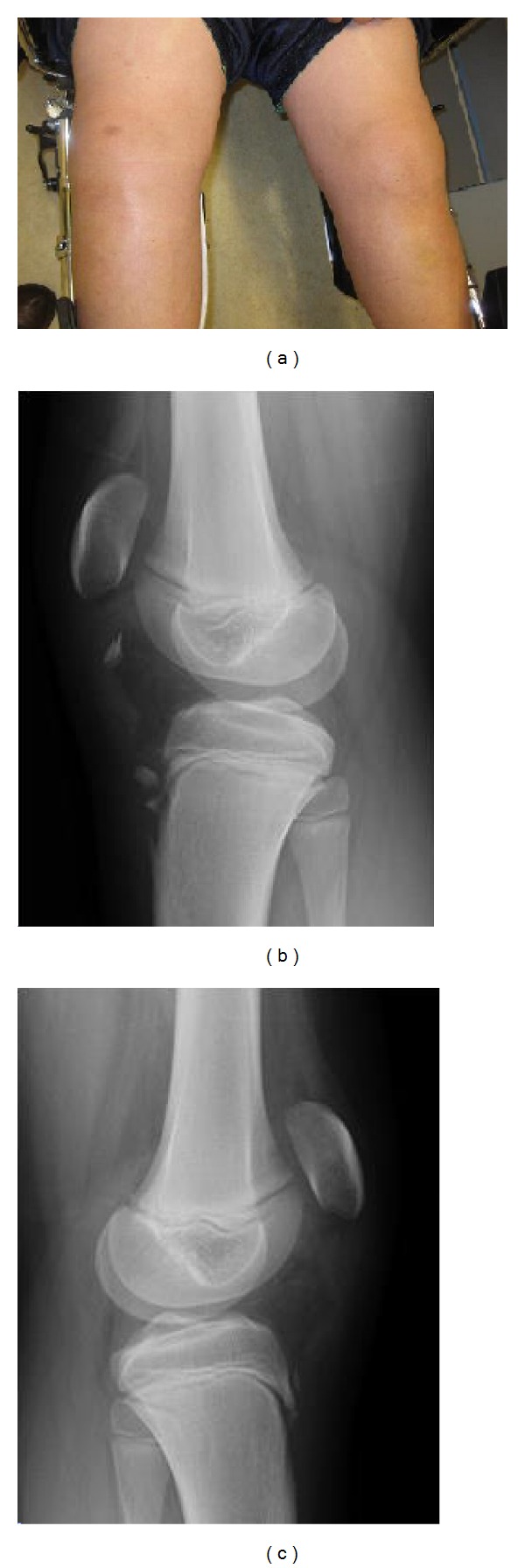
Clinical photograph shows bilateral anterior knee swelling. Bilateral lateral knee radiographs demonstrate the flecks of bone and high-riding patella. Patellar tendon soft tissue shadow shows the waviness of the tendon indicating loss of its integrity.

**Figure 2:**
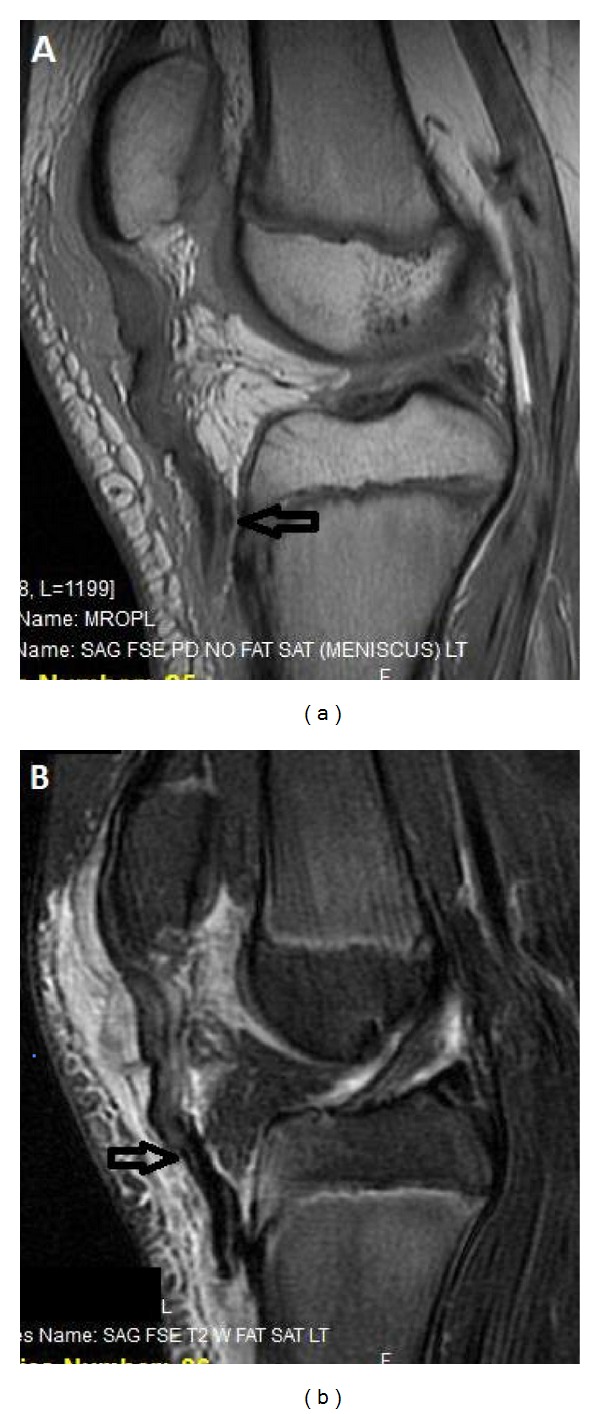
(a) Left knee MRI shows patellar tendon avulsion with the arrow pointing to the small bone fragment. (b) MRI demonstrates patellar tendon avulsion from its distal attachment on the right side. The arrow marks the distal extent of patellar tendon with the periosteal flap attached distal to it.

A 12-year-old Caucasian boy presented to orthopaedic clinic with an acute injury to both his knees. While running during football practice, he tried to stop abruptly and felt sudden pop in both his knees. His feet were planted at the time of injury. He fell on the ground with complaints of severe pain in both knees and inability to bear weight on his legs. The patient and his family denied any history of systemic disease, chronic medications, or history of Osgood-Schlatter disease. On physical examination, the patient had severe diffuse swelling around both his knees (Figure 1). The patella was high-riding and he was unable to perform any active range of motion of his knee. Any attempt to move his knee was painful. Bilateral knee radiographs showed small flecks of bone near the tibial tubercle on the right side and a small avulsion fracture of the tibial tubercle on the left side (Figure 1). There was patella alta on both sides with the Insall-Salvati index being 1.6 on the right side and 1.5 on the left. Bilateral knee magnetic resonance imaging (MRI) confirmed the diagnosis of sleeve avulsion fracture of the tibial tubercle. It demonstrated the patellar tendon being pulled off the anterior tibial metaphysis with large periosteal flap and small bone pieces on the right side and patellar tendon avulsion with a small bone piece and periosteal flap on the left side (Figure 2). On both sides, there was partial tear of patellar insertion of patellar tendon. 

**Figure 3:**
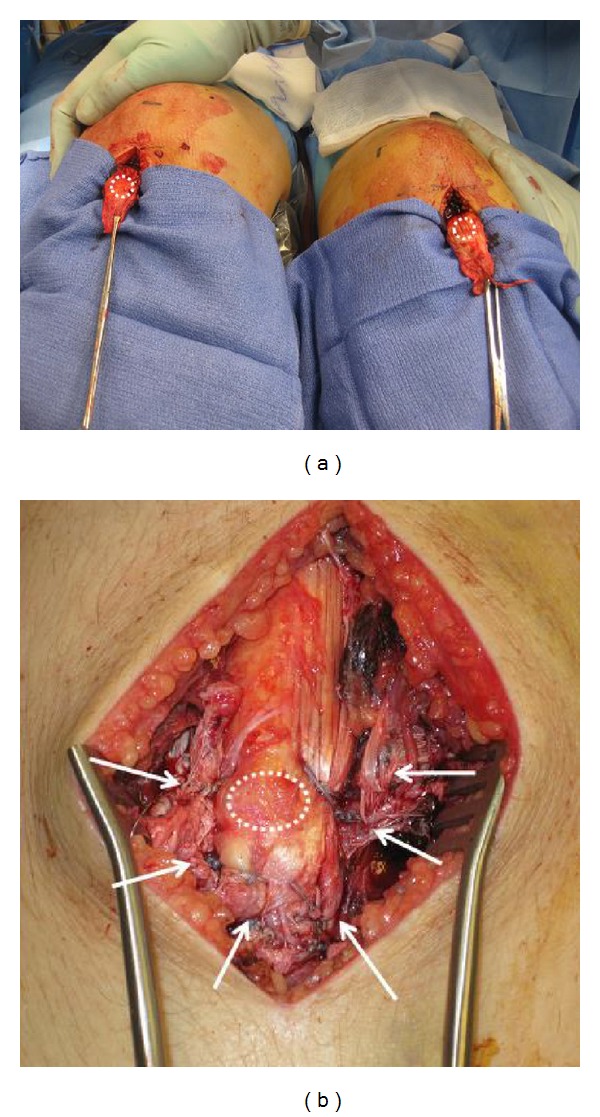
Intraoperative images show an anterior approach to bilateral patellar tendon sleeve fractures. The dotted circle is the distal attachment site of the patellar tendon to the tibial tubercle. Soft tissues distal to the circle are the periosteal flaps attached to the patellar tendon which are repaired to the surrounding soft tissues (arrows).

At surgery, the patellar tendon was repaired to the tibial tuberosity using a 5.5 mm BioCorkscrew suture anchor, preloaded with two No 2 Fiberwire suture (Arthrex, Naples, FL, USA). The periosteal flap was sutured back to the area of its avulsion using two 5.5 mm BioCorkscrew suture anchors, one on each side of tibial tuberosity (Figure 3). Similar 3-anchor repair was performed on the opposite knee. The repair was performed with the knee in 30° of flexion, and the height of the patella was confirmed on lateral fluoroscopic image using intercondylar notch line as reference. 

**Figure 4:**
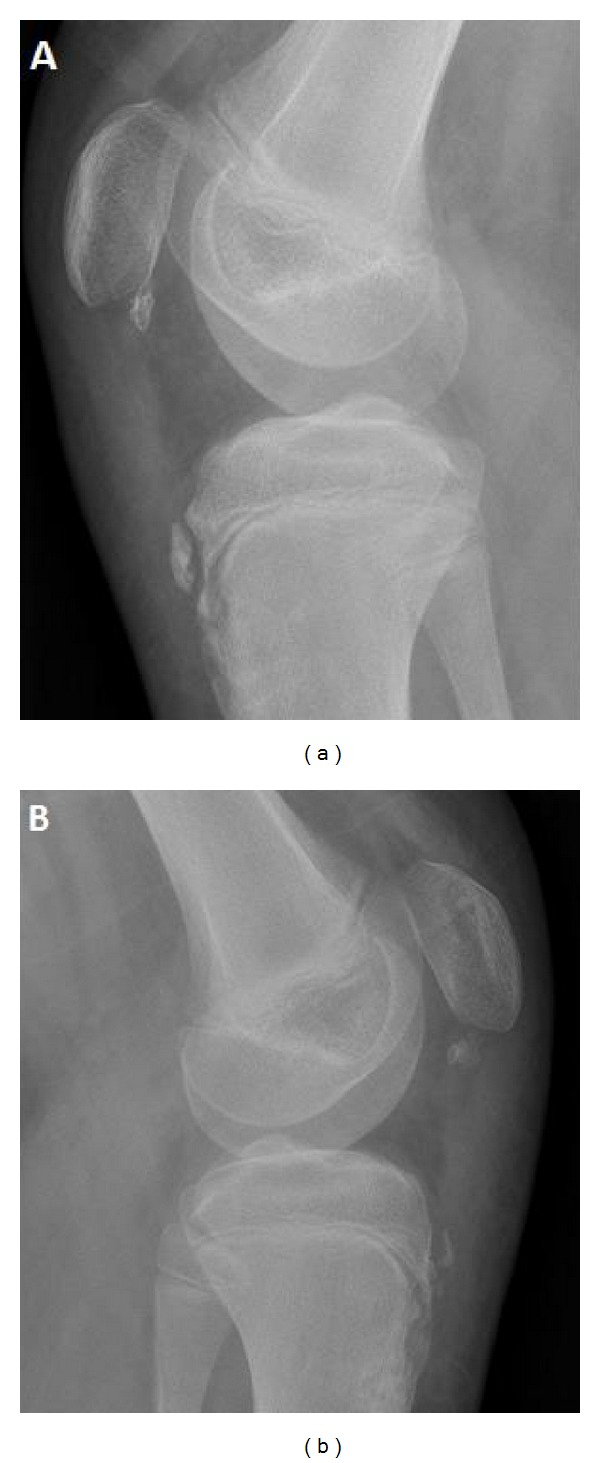
Postoperative radiographs of (a) right and (b) left knees show a decrease in patellar height.

**Figure 5:**
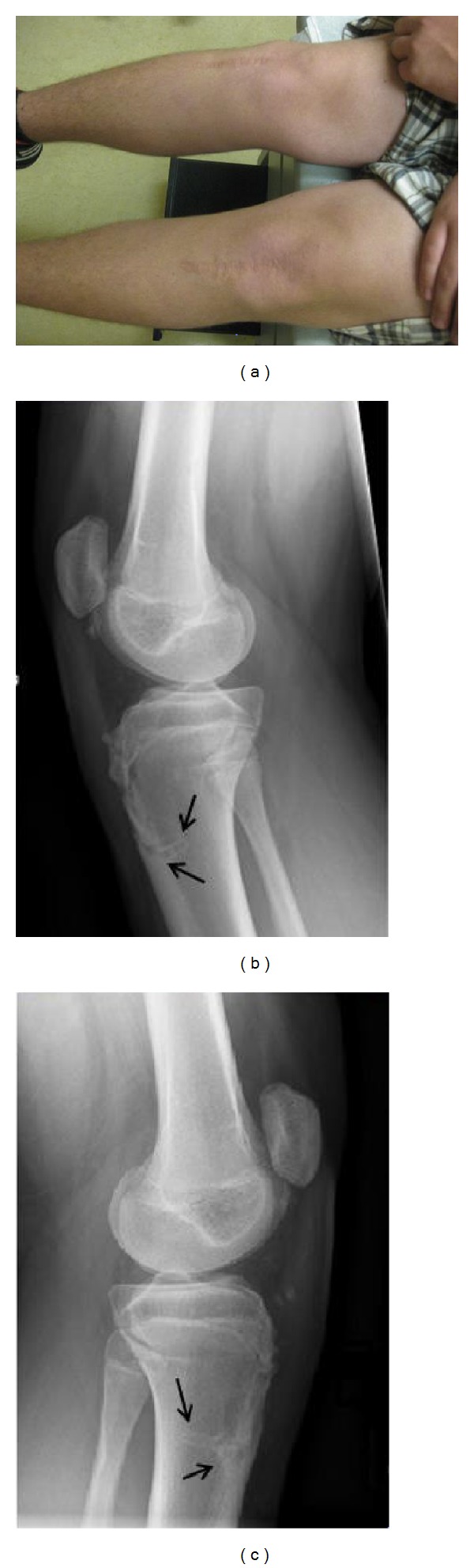
At three-year followup, the patient has full range of active motion and no strength deficits for knee extension. Lateral radiographs show irregularity in the area of patellar tendon repair, sites of suture anchor placement (arrows), and maintenance of posterior tibial slope without any growth disturbances.

Postoperatively, the patient was placed in bilateral hinged knee brace locked in extension. Physical therapy was started one week after surgery with gradual passive range of motion and static quadriceps exercises. Postoperative radiographs at 2 months showed a decrease in the patellar height with an Insall-Salvati index of 1.3 on the right side and 1.2 on the left side (Figure 4). Radiographs of both knees also showed evidence of injury at the patellar attachment of patellar tendon. Physical therapy was advanced to gain full range of motion by the third postoperative month. After 4 months, the patient had adequate strength in his quadriceps muscles and home exercises were recommended. When evaluated three years after surgery, the patient did not have any functional deficits related to his lower extremities (Figure 5). Radiographs showed no growth disturbances (Figure 5). 

## 3. Discussion

 The failure of patellar tendon is relatively uncommon amongst extensor mechanism injuries. Zernicke et al. [4] described that it requires 17.5 times the body weight to rupture the human patellar tendon. The common mechanism of injury is simultaneous violent contraction of quadriceps muscles against forceful flexion of the knee. The rupture can occur either in the intratendinous portion of the tendon or at its attachment at the osseotendinous junction. Bilateral rupture of the patellar tendons is rare. The changing mechanical properties of the osseotendinous junction with age and the rate of deformation (slow stretch versus fast stretch) may be responsible for the differences seen in these varied injury patterns [5].

There are a few reported cases of simultaneous bilateral patellar tendon rupture in children. The rarity of such injuries can be attributed to the fact that these injuries require a high magnitude of force which the kids are rarely exposed to. These patellar tendon ruptures can occur in its midsubstance or as sleeve avulsions from the patella or the tibial tubercle. Muratli et al. [6] described a case of simultaneous bilateral midsubstance rupture in a 9-year-old boy following a fall. Kim et al. [7] described a case of 12-year-old boy with bilateral simultaneous avulsion of patellar tendon from inferior pole of patella which required surgical intervention. 

Sleeve fractures represent avulsion of thick periosteum and cartilage with flecks of osseous structure. It has been previously described in capitellum [8] and patella [2, 3]. Houghton and Ackroyd [9] was the first to describe patellar sleeve fracture in children in 1979. In 2002, Davidson and Letts [1] reported a series of three patients with unilateral sleeve fractures from tibial metaphysis. Our case is unique in that it represents the first case of bilateral tibial tubercle sleeve fractures. None of the available classification systems for tibial tubercle fracture in children include sleeve fractures. It is recommended by previous authors that tibial tubercle sleeve fractures should be added to the existing classification system for tibial tubercle fractures as a type 5 fracture in the Watson-Jones system [10] and as a type 4 fracture in the Ogden classification system [11]. 

The diagnosis of these injuries could be challenging. Physical examination is critical to an accurate diagnosis. High-riding patella, palpable gap at the site of patellar tendon, and an inability to perform active extension are important findings suggestive of patellar tendon injuries. At times, swelling, pain around the knee, and guarding can make clinical diagnosis difficult. Lateral knee radiographs should be carefully evaluated for patella alta or small flecks of osseous fragment near the tibial tubercle which can aid in diagnosis. MRI can confirm the injury pattern and can be helpful to evaluate the size of the sleeve. The sequelae of a missed diagnosis or inappropriate treatment of extensor mechanism disruption could be devastating including severe extensor lag and restricted knee flexion [12].

Treatment of the tibial tubercle sleeve fractures is different from tibial tubercle fracture. Since tibial tubercle sleeve fractures are mainly periosteal injuries with tiny pieces of bone, they can be treated with small screws with suture supplementation. In the three cases reported in the literature, two cases were treated with small cancellous screws and periosteal sutures, whereas one case was treated with sutures alone. Our patient was treated with nonabsorbable suture fixation (and not screws) due to the small size of bone pieces and large periosteal sleeve fragment. The final outcome was excellent in the presented case.
